# The impact on nurses and nurse managers of introducing PEPFAR clinical services in urban government clinics in Uganda

**DOI:** 10.1186/1472-698X-11-S1-S8

**Published:** 2011-03-09

**Authors:** Joyce Nankumbi, Sara Groves, Elli Leontsini, Nambusi Kyegombe, Alex Coutinho, Yuka Manabe

**Affiliations:** 1 Department of Nursing, School of Health Sciences, College of Health Sciences, Makerere University, Kampala, Uganda; 2 Johns Hopkins University School of Nursing, Baltimore, Maryland, 21205, USA; 3 Johns Hopkins Bloomberg School of Public Health, Baltimore, Maryland, 21205, USA; 4 London School of Hygiene and Tropical Medicine, London, UK; 5 Institute of Infectious Disease, Kampala, Uganda; 6 Johns Hopkins School of Medicine, Baltimore, Maryland, 21205, USA

## Abstract

**Background:**

Improving provider performance is central to strengthening health services in developing countries. Because of critical shortages of physicians, many clinics in sub-Saharan Africa are led by nurses. In addition to clinical skills, nurse managers need practical managerial skills and adequate resources to ensure procurement of essential supplies, quality assurance implementation, and productive work environment. Giving nurses more autonomy in their work empowers them in the workplace and has shown to create positive influence on work attitudes and behaviors. The Infectious Disease Institute, an affiliate of Makerere University College of Health Science, in an effort to expand the needed HIV services in the Ugandan capital, established a community-university partnership with the Ministry of Health to implement an innovative model to build capacity in HIV service delivery. This paper evaluates the impact on the nurses from this innovative program to provide more health care in six nurse managed Kampala City Council (KCC) Clinics.

**Methods:**

A mixed method approach was used. The descriptive study collected key informant interviews from the six nurse managers, and administered a questionnaire to 20 staff nurses between September and December 2009. Key themes were manually identified from the interviews, and the questionnaire data were analyzed using SPSS.

**Results:**

Introducing new HIV services into six KCC clinics was positive for the nurses. They identified the project as successful because of perceived improved environment, increase in useful in-service training, new competence to manage patients and staff, improved physical infrastructure, provision of more direct patient care, motivation to improve the clinic because the project acted on their suggestions, and involvement in role expansion. All of these helped empower the nurses, improving quality of care and increasing job satisfaction.

**Conclusions:**

This community-university HIV innovative model was successful from the point of view of the nurses and nurse managers. This model shows promise in increasing effective, quality health service; HIV and other programs can build capacity and empower nurses and nurse managers to directly implement such services. It also demonstrates how MakCHS can be instrumental through partnerships in designing and testing effective strategies, building human health resources and improving Ugandan health outcomes.

## Background

The global deficit of trained health workers in Africa is estimated to be more than 4 million, and the Global Health Worker Force Alliance estimates that 1.5 million new workers need to be trained to address the current shortfall in Africa’s health systems [1]. With this deficit much interest has recently surrounded how to streamline HIV care, both to offer high quality care to patients and expand access to care with fewer workers. In countries severely affected by HIV/AIDS, shortages of health workers including qualified health care professionals present a major obstacle to scaling-up HIV services. Health services, as a labor intensive activity, depend ultimately on the performance of the workers, and improving their performance is central to the building of health capacity and should receive high priority.

Nurses are the largest group of health care providers, and it is essential that these nurses feel engaged in their work, have a satisfying work environment and thus experience job satisfaction. Giving nurses more autonomy in their work empowers them in the workplace and has shown to create a positive influence on work attitudes and behaviors [2,3]. Laschinger and colleague, using a model of structural empowerment, argue that for nurses, access to information, access to support, access to resources needed on the job, and the opportunities to learn and grow will increase job satisfaction, commitment, and productivity, with fewer adverse patient events [2]. Empowering nurses will make them more effective health providers.

Makerere University College of Health Sciences (MakCHS) in Uganda is undergoing a major organizational transformation to more effectively impact the health sector in Uganda and internationally. The improvement of health provider performance has emerged as a high priority. As a center of learning the University provides an environment where new ideas and new models of health care can be generated to meet the health worker crisis [4]. As part of meeting the grander challenge of building innovative systems to sustain better health in Uganda, a community-university partnership was created to demonstrate how to better serve the HIV needs of the disadvantaged population of Kampala City.

In the Ugandan capital, the Kampala City Council (KCC) health division is mandated by the Ministry of Health (MoH) to be the local provider of services, including primary health care to local city residents. The council administers a total of eight nurse managed health centers (HCs) distributed throughout the city to meet this mandate. Mulago-Mbarara Teaching Hospitals’ Joint AIDS Program (MJAP) in 2005 received supplemental funding to pilot a program to provide comprehensive HIV treatment in two of the KCC clinics. By expanding these two clinics they had the potential to deliver HIV services closer to the community, and to improve access to care. At the same time, this enabled referral centers like Mulago National Referral Hospital and its associated clinics to decongest and actually operate as referral units and not as a primary health care site. The pilot program consisted of providing supplies and equipment, training, and trained physicians that would assist in the clinics once weekly.

With the success of the pilot program, the potential of using all the clinics to deliver PEPFAR funded HIV prevention, care, and treatment was discussed between the Infectious Disease Institute (IDI), an affiliate of the Makerere University College of Health Sciences, and the responsible government authorities. In 2006, IDI and MJAP partnered with the MoH to build the capacity of the six KCC clinics to deliver HIV/AIDS care.

In the development of this program IDI adapted and exported their best practice model of HIV care delivery to the KCC clinics which included: routine HIV testing and counseling (RTC), HIV basic care and psychological support, TB diagnosis and treatment, antiretroviral therapy (ART), and expanded laboratories. IDI also implemented capacity building activities in these six nurse managed KCC urban health centers, including training and technical assistance to staff for clinic and pharmacy management, strengthening clinic management capacity to monitor patients on ART, and providing the nurse managers the skills in procurement and supply chain for drugs and supplies. Over 25,000 individuals in the six KCC clinics received HIV counseling, testing, and treatment as needed between 2006 and 2009. The program was a success from the PEPFAR viewpoint having achieved expansion of RTC services and HIV treatment via community-based care.

One of the objectives of a MakCHS and Johns Hopkins University (JHU) learning grant was to develop and test effective strategies for the University and the community partners to use to improve health services and outcomes related to national health priorities [4]. The grant provided an opportunity to evaluate the KCC project from the point of view of the University mission, to demonstrate how MakCHS could play a critical role within the Ugandan health system. This paper presents the impact of introducing HIV PEPFAR clinical services into local, nurse-managed, urban, government clinics in Kampala, Uganda. The study was initially designed to evaluate the influence of this community-university partnership on the KCC nurse managers and staff that work at these clinics. The evaluation framework asked the nurses about their knowledge of the key project initiatives, partner involvements at their clinic, capacity building, job satisfaction, effect on services and the challenges of the new program. This paper provides information on this new model of care, and examines the model’s effectiveness as perceived by the nurses in this low resource setting.

## Methods

A mixed method approach was used. The study was descriptive, collecting both qualitative and quantitative data between September and December, 2009. Key informant interviews were conducted on site in English by two of the authors with the nurse managers of each of the six KCC clinics (Kisenyi, Kiruddu, Komamboga, Kiswa, Kitebi and Kawaala) in Kampala. In addition a structured questionnaire in English was administered by a research assistant to all the staff nurses working at the six clinics on the same day as the key informant interview. In total data was collected from 20 staff nurses (all the nurses who qualified consented) and the 6 nurse managers.

The Key Informant (KI) interviews followed a semi-structured guide to explore the experiences of the nurse as a manager, their roles in the clinic, the challenges, and the opportunities as a result of the PEPFAR program being placed in their clinic. The KI interviews were audio recorded with the permission of the nurses and detailed notes were taken at the time of the interview.

Each KI interview lasted about one hour. All interviews were transcribed, providing a full record of each of the interviews. The transcripts were reviewed by the recorder (interviewer) for accuracy. The interviews were then read by two member of the research team, manually analyzed and jointly summarized. During the analysis, key themes were identified in the transcripts, focusing on issues that were mentioned frequently or consistently, that received particular emphasis, or for which KI views expressed in the interviews diverged in a systematic way. The textual data was structured in matrices, with column for different thematic areas, and rows that illustrated the themes. This approach facilitated comparison across and within groups. The other members of the research team reviewed the themes identified and provided comments.

**Figure 1:**
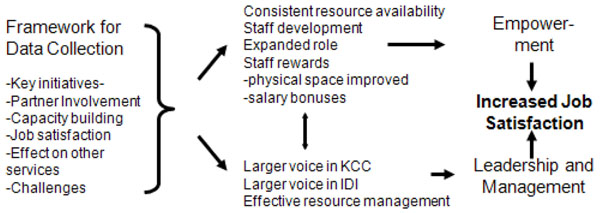
Framework for data collection and data analysis

Based on the information in the KI interviews, the themes of empowerment and leadership emerged (see figure 1). Empowerment was defined as facilitating the nurses to think, take action and control work and autonomous decision-making. The areas that facilitated empowerment included access to information, access to support, access to resources needed on the job, the opportunities to learn and grow, and increased in confidence and autonomous nursing practice [5]. Under the theme of leadership and management the tasks of planning, providing staff direction, monitoring of the operations and representation with administration were included as nurse manager skills [6]. This framework was then used to analyze the data.

After receiving written consent the staff nurse questionnaire was administered by a trained research assistant and supervised by the researchers doing the KI interviews. The questions asked the nurses about their work experience at the KCC clinics, the shift in work load, impact of new services on the other clinic services, any training they received as part of the new project, the current perception of their new work experience and their perception of the IDI team working at the clinic. This questionnaire was created for assessment purposes and followed a similar framework as the KI interviews. The 45 question survey took about 30 minutes to complete. The data were reviewed for completeness, entered into windows excel program and analyzed using SPSS version 15.

The research was approved by the Institutional Review Boards of the College of Health Sciences Makerere University and Uganda National Council of Science and Technology. Johns Hopkins School of Public Health IRB also assessed the project and determined that no additional approval was required.

## Results

### Nurse managers

The six nurse managers that participated in the key informant interviews were all women between the ages of 35 and 45 years and were double trained with diplomas in both nursing and midwifery. The duration of their service as clinic manager ranged from six months to six years. Their designated role as clinic manager included supervision and coordination of the different clinic activities, ensuring the cleanliness of the clinic, attendance at monthly administrative meetings, report writing, and requisitions of the essential supplies (including pharmacy) and equipment for the clinic. The managers also collaborated with different partners interested in working with the clinic. In addition, several of the nurse managers supervised nursing and other health science students in research and community-based education. The nurse managers were responsible for supervising the clinical staff and were also involved in provision of in-service education and skills training for nurses and other health care providers. The number of clinic staff (including all health care providers and support staff) at each clinic varied with a range of 24 to 40 staff with a mean of 32.

### Leadership and management

#### Opportunities for management and leadership

Clinic managers were asked what they knew of the key initiatives of the project. The majority understood the project and recognized the over-arching goals of reducing patient congestion at Mulago, decentralizing HIV care services, and equipping other persons/health care providers in HIV care through knowledge and support. The managers also recognized and implemented the need for expansion of the Voluntary Counseling and Testing (VCT) services, tuberculosis (TB) care, and offering ART and treatment for opportunistic infections. They utilized improved infrastructure, equipment, and clinical support, and key initiatives of the project to improve clinic services. The managers expressed on several occasions that the partners valued their managerial skills as the sister-in-charge, gave them respect, listen and acted on their contributions, but also put more administrative demand on them in supporting the new clinic.

With the increase in patient numbers there were more administrative duties, more supplies to order, new clinic budgets to consider, the accountability of more funds received, and allocation of these funds to specific projects/ clinics. The nurse managers also had new IDI/KCC project duties. As the clinical administrator for the project, they needed to ensure there were adequate stocks of medicines and sundries, patients were seen on the specific project days, data for clinic services was collected, and information was compiled into a monthly report. The nurses said that although there was more work it didn’t substantially increase their work load because they were equipped with new knowledge, skills training, and the logistical system to do the tasks.

A nurse manager described the leadership the nurses took in developing a community program.

“The nurses motivated the Princess Club, and they got 4 active members from the HIV positive patients and they trained them as peer counselors, and these people can go to the community, they can follow up at the clients within the community, they can train, they can counsel and they are also given allowances, they are paid. It has also led to behavior change, when they come here they are counseled, they are taught how to behave.”

The stock-out of non-HIV drugs to treat malaria and infections was a big challenge. One nurse took on a leadership role and went to the district MoH and persuaded them to increase the funding for the medications. She was able to get more drugs, but some stock-outs persisted.

#### Planning

The nurse managers had not been stakeholders in the initial partnership and planning by IDI and the MoH. However, as the partnership developed the nurse managers were actively involved in the new clinic collaboration. The nurses saw this as an opportunity to participate in better planning for health care services and outcomes for the entire clinic due to the availability of a greater quantity and quality of supplies and equipment.

The nurse managers mentioned the following as part of the project objectives and planning components in which they participated:

“We can plan how to move medications that are about to expire from one clinic to another or to Mulago to avoid drug wastage with the help of our partners.”

“We have with the grant, planned for improved space and new infrastructure to improve waiting areas for patients, increase the number of consultation rooms, and have more bathrooms.”

As a result of actively participating in the planning the nurses noted that

“We can provide more comprehensive care to HIV patients: expansion of voluntary testing and counseling services, provide community-based care and at the same time decongesting Mulago IDI, expansion and improvement of TB care, support ARV home care, and the treatment of opportunistic infections.”

“I purchase drugs and do the accountability,” one nurse manger reported. “We have more consistent supply of medication.”

Another nurse manager stated, *“We have more equipment to better exam HIV patients, for example laboratory equipment, BP machines.”*

“We can have continuous HIV in-service education to teach staff how to treat, identify cases, and counsel clients.”

#### Monitoring the operations

The managers stated that with improved infrastructure patient care services by the nurses and laboratory technicians had improved significantly. This made it easier for the nurse managers to monitor and improve the operations. They were able to collaborate with partners to hand out basic care kits to HIV patients that included tuberculosis drugs and drugs for opportunistic infections. They were able to use test kits to screen the community, and then provide service to the HIV positive clients. With the improvements of supplies and management of drugs and other daily consumables they stated they provided better service. The managers pointed out that the partners were even conscious of making the operation run smoothly by making sure there were such things as tea, sugar and toilet paper for the MoH staff.

#### Representation with administration

The mangers reported having regular staff meetings and they also attended health facility management at the district level. They were very happy that they were always included in all the meetings occurring within the project, and that their opinion was valued. They were included in the decision making of their own clinics and were consulted on issues like staffing, supplies, changes in infrastructure, and inservice education.

“The system of working with the partnership is good. When there is a challenge it is discussed and they [the partners] look immediately for a solution or an alternative.”

### Empowerment

#### Staff rewards with physical space

Before the partnership the large patient numbers and lack of equipment was a challenge that had been impossible to address with overcrowding, no space for consultation, having to refer for laboratory work, and lack of medicine. The managers said that at the beginning of the partnership their space was even more limited due to the large influx of HIV patients. Before the renovations were completed and the clinics reorganized, the KCC clinics with the larger patient populations were not physically able to provide all of the previous services (e.g. immunizations, and antenatal services). With the completion of the improved infrastructure all the nurses reported that they could now provide these services. The improvement of infrastructure included patient and staff toilet facilities, pharmacy storage, increased consultation rooms, laboratory upgrading with equipment (CD4 machine, HIV testing kits, sterilizing equipment), and increased supplies (e.g. needles, gloves).

The nurse mangers commented:

“They[MJAP-IDI]recently constructed a big shelter where the client can sit, while waiting for the doctors, and can be counseled from there, they constructed a counseling rooms, doctor’s rooms, they provided chairs, tables, TV screens, exam table and many other things”.

“With City Council we couldn’t, but with IDI, we usually sit in a meeting and then present our problems, so they managed to provide the necessary infrastructure and human resources.”

The managers also mentioned that their operations could also now extend directly into the community. With support from the partners they now had the resources.

#### Salary bonuses

The managers stated that nurses at the clinics benefited from several rewards. They reported that all the nurses received an additional allowance as a motivation for the project work or the extra regular clinical work done; this significantly improved everyone’s morale. A few of the nurse managers pointed out that although everyone received an additional allowance not everyone had project responsibilities, creating some job dissatisfaction for those with the additional project work. For example, the nurses in the HIV clinic stated they had new project work and they wanted more money than their colleagues not working in the HIV clinics received. Likewise, a few of the staff nurses said they didn’t understand why all nurses got additional money, although the managers understood completely. However, most of the staff was happy with the additional income, agreeing that they all had additional work. The nurse managers reported that none of the staff were de-motivated, but everyone was concerned with what would happen if/when MJAP/IDI pulled out of the project.

#### Staff development

The managers recognized that human resources were an essential input in the delivery of better health services. The managers were pleased that the staff received new training in HIV care, service provision, and logistics management. This led, according to the managers, to increased patient services, better HIV counseling, and increased opportunity for patient behavioral change. The managers listed several inservice trainings as a result of the partnership: staff management workshop, ART care and medications, prevention of mother-to-child transmission (PMTCT), pediatric HIV management, palliative care, data management, counseling, multidisciplinary workshop on HIV care, and human resource management. Many of these topics were particularly relevant to the nurse managers in their extended roles as a result of task expansion in the clinic. The managers said an additional benefit of the training was that at the continuing education programs they developed collegial relationship across the clinics allowing them to share different challenges and solutions.

Expanded tasks of both the nurse managers and the nurses included: review and management of HIV patients on their clinic days, management of opportunistic infections in these patients, and referral for patients with complications. They also provided initial medical care to the patients with newly diagnosed HIV.

“We no longer send away HIV patients even if it is not a clinic day, we are able to manage them.’’ Nurse Manager

The nurse managers stated:

“The training was beyond the HIV clinical care. Provided information in data management”

“Have had several training: human resource management, effective management, logistic management, malaria management, taught them about HIV drug management (nurse can now prescribe), nearly all staff received training at IDI, trained in mother to child transmission prevention, palliative care”

“The training has really done good because a mere single registered nurse and midwife, if they don’t go for these workshops; you never know what to do. For example I can now start a patient on ARV’s… and how to manage side effect. In case we are to operate alone you know what to do”

The managers were especially cognizant of the increase in clinic capacity because of the increase in knowledge among the staff.

*“We have the skill to now manage HIV patients even if it is not the project day. We don’t turn the patients away.”* Nurse manager

*“I must say that they have really built capacity in this clinic, we are able to at least do good work, I must appreciate that the coming in of this project, I think we have become better people now.”* Nurse manager

*“Maybe for antenatal, we didn’t have the PMTCT services but we initiated the PMTCT services, this was because people were not trained in provision of the PMTCT services but later on midwives were taken for training and we started the service.”* Nurse manager

### Expanded role

The nurse managers reported that there was not much change in their work load or role, and actually they were now better equipped to meet the challenges of a busy clinic. One nurse manger reported that the project collaboration runs very smoothly and needs little of her time.

“I do not just relate now to KCC but also I am the clinic administrator for this project, I was given management training and a multidisciplinary HIV workshop.”

Some managers reported added responsibility: increased paperwork in monthly reports, more accountability for medicine, spending more time in management, supervising of staff and patients, and more work with data entry. However, another nurse noted that with increased self reliance among the staff,

“I have to spend less time in general OPD on the day of the clinic”

When asked: Is the role harder with the new clinics? The nurses replied**:**

“No, it is not harder, but a little heavier because of the additional work.”

“No, not really. You feel you are engaged from time to time, am busier than I was.”

“No, my work is easier. There has been many staff trained, so I am not working alone but have a team. We integrate and work together.”

### Challenges faced by nurse managers

The challenges identified from introducing the project included: limited space, client overload, clinic understaffing, poor hygiene, lack of skills in handling large number and different patients, and stock outs for drugs such as antibiotics and anti-malarials.

*“Client overload means everything must be doubled but you find that despite the increasing numbers, certain things have remained constant so meeting the gap is still a big challenge.”* Nurse manager

The challenge of space is one of the biggest.

*“Client congestion is a problem, cleanliness of the compound. So at the end of the day you find the compound full of papers, full of empties of drugs, but we try our level best to clean at the end of the day. We are also overwhelmed because the temporary closing of a clinic to restructure the old clinic.”* Nurse manager

Too small a pharmacy and poor space for distribution were complaints in one clinic. The new program worked with the staff to change the infrastructure, and the nurse reported they now had new areas for drug storage, a redesigned pharmacy distribution to one location, and an increased patient flow which at the same time reduced stigma of HIV medication being given in a different location. Additional challenges included inadequate staff in one clinic.

### Staff nurses

Interviews were carried out with the clinical staff nurses to explore the impact of the HIV PEPFAR services on their work experience, job satisfaction, workload, views on other clinic services and their in-service training. Twenty nurses were interviewed from the six different clinics (Kiruddu, Kisenyi, Kiswa, Kitebi, Komamboga and Kawaala). They described their current job as including counseling, drug dispensing, general clinic nursing care, and specific HIV and TB care.

Of the 20 staff nurses interviewed 70% had worked at the clinic for more than 1 year, and they liked the job because of opportunities to improve their skills with additional training (Table 1). The majority of the nurses reported that after the project started they had spent more time with the patients; job satisfaction had improved as a result of the project initiation.

**Table 1:** Clinic Experience and Job satisfaction

Time worked at clinic		Like most about clinic		Like least about clinic	
<6 months	1 (5%)	Distance from home	5 (25%)	Distance from home	2 (10%)
6 to 12 months	5 (14%)	Can respond to patient needs	6 (30%)	Salary	7 (35%)
>1 year	14 (70%)	Opportunity to improve care	9 (45%)	Facility	4 (20%)
				*Other	7 (35%)

*Others: no opportunity to improve technical skills, too few staff, to heavy workload, poor working conditions

Eighty percent (n=16) of nurses reported having more work to do after the initiation of the project, with only 5% (n=1) reported having the same amount of work before and after the project started. Over half of the nurses (n=11, 55%) reported having more time to spend with the patients after the restructuring of the clinic. Four of the nurses (20%) stated that they now spent less time with patient, three nurses (15%) the same amount of time, and 2 nurses (10%) were not sure if they spent more or less time with the patients.

The nurses reported that all services had improved with the exception, in some of the clinics, of pediatric care (Table 2). Of the nurse respondents, 35 % stated there was a decrease in pediatric services. This could in part reflect the initiation of a new pediatric HIV center sponsored by Baylor College of Medicine providing care to the same population near the KCC clinics, decreasing the utilization of the KCC services.

**Table 2:** Effect of the project on other KCC clinic services

Services	Services improved	Services are same	Services worse
Antenatal care	17 (85%)	3 (15%)	0
Immunizations	13 (65%)	7 (35%)	0
Pediatric care	2 (10%)	11 (55%)	7 (35%)
Dental care	8 (40%)	12 (60%)	0
Other non-HIV care	10 (50%)	10 (50%)	0

Ninety percent of the nursing staff that was included in this study had received training from IDI in HIV and non-HIV areas. As summarized in Table 3, 95% of those that received this training felt that the training had been useful to their current work, and that through this training they had acquired both clinical (70%) and non clinical knowledge and skills (55%). The training also allowed 60% of the nurses to refresh knowledge and skills previously learned.

Table 3 presents the perception of the nurses about their work experience using a 5 point Likert scale were 1 indicated the lowest level of agreement and 5 indicated the highest level of agreement with a given statement. The nurses responded positively about the effect of the clinic restructuring on their work experiences, especially in the areas of their confidence in carrying out their work, feeling valued, having received adequate training, and providing quality care. The lower rating had to do with lack of the best environment to provide the care. They did not find the clinic highly stressful.

**Table 3:** Nurses 5-point Likert scale responses to perception of KCC work experiences after the clinic restructuring.

Statement	N	Mean [Range]
Feel supported in carrying out my work at the clinic	20	4.05 [2-5]
Feel confident when carrying out my work at the clinic	20	4.65 [4-5]
Feel the work I do is important and well valued	20	4.55 [2-5]
Find the work load at the clinic is too demanding	20	3.3 [2-5]
The facilities at the KCC clinic are adequate for me to carry out my work	20	2.65 [1-5]
Work at KCC is stressful	20	2.3 [1-5]
Training that I have undertaken has improved my skills	20	4.7 [4-5]
Training has improved the knowledge	20	4.4 [4-5]
Quality of care I provide has improved	20	4.6 [4-5]
Each patient is given the right amount of time	20	3.45 [2-5]

## Discussion

Like much of Africa, provision of more universal HIV services in Uganda created extraordinary health systems demand that could not be met using traditional physician dependent models. In the context of this PEPFAR-funded community-university partnership emphasis was placed on utilizing every available human resource to its full potential. The three-pronged approach of training, provision of necessary resources, and continued support allowed the rapid integration of services and care despite some notable human resource constraints. Both the nurse managers and the staff nurses found that the training and support to the nurse cadre helped to improve the services delivered to patients under this newly structured program. Our data agree with others who found that good health outcomes can be achieved by enhancing the role of the nurse [8,9].

The primary intent of this study was to evaluate the impact of introducing PEPFAR clinical services into the local KCC urban government clinics. In actuality it identified the effectiveness of empowering nurse in their role of providing primary care as they scale up the quality of care provided HIV patients as well as other services provided. Two of the largest dilemmas experienced universally by nurse managers are having problems that appear impossible to solve due to a lack of adequate resources, and individual staff apathy [10]. Effective provision of resources is key to the empowerment of staff and essential for work effectiveness, for nurses to function autonomously, for patient’s safety and quality care, and to promote collaborative/collegial relationships. According to the nurses, this project provided those resources in a well-managed approach that helped solve some of the insoluable problems and immediately engaged staff interest.

With triangulation of the data from the nurse managers and staff nurse, the responses reflected similar views and perceptions. All of the nurses agreed that the increase in training provided new skills and knowledge that could be immediately implemented in the direct service to the patients at the clinic. Immediate implementation of the new information is likely to increase the trainees’ interests and efforts [11]. The nurses also agreed that the care was improved and that specifically the staff nurses had more time to spend with the individual patient. The improvement in infrastructure and particularly increased lab capabilities, more space, and reliable sources of medications with no stock outs were also named as important developments under the project. Improvement in the nurse’s work environments in health centers has the potential to empower the nurses, simultaneously reduce high level of job burnout and decrease the risk of turnover [2,12]. It also increases job satisfaction [13] and there has been increased patient satisfaction with care at the KCC clinic [14]. The staff nurses, however, were in agreement that they had more work with the restructuring, even though the nurse managers felt the work load had not increased. Although, in general, the nurses were very satisfied with the collaboration, there was some concern that pediatric care decreased in a few of the clinics; an issue that warrants further exploration.

A skilled nurse manager can be a huge asset to the effective functioning of a clinic, and actions that can be taken to enhance any of the components of an effective manager will be reflected in the provision of better health care. According to Tappen there are seven components to an effective nurse manager: leadership, planning, providing staff direction, monitoring the operations, distributing fair rewards, staff development, and representation with administration [6]. The nurse managers in their interviews volunteered all seven of these components as actions that were enhanced as part of the partnership, and the nurses considered each component as an important part of a successful improvement of the clinic nurse manager role. They were appreciative of the extensive management training that they received that equipped them with the skills, knowledge, and competencies to carry out their roles. The nurse managers by participating in the partner meetings had knowledge of organizational decisions giving them the ability to make decisions that contributed to the organizational goals.

Research indicates that low levels of job satisfaction are prevalent among nurses and this poor satisfaction leads to job turnover. Sources of low satisfaction are associated with factors that interfere with patient care, feeling overloaded with work responsibilities, poor relations with co-workers, personal factors, organizational factors, and a lack of power in the job setting. Nurses’ ability to moderate aspects of the work environment correlates with job satisfaction [15,2], and in this project, the nurses repeatedly talked about how they modified and improved their environment through provision of necessary drugs and supplies on top of the training received.

The difference in satisfaction levels is also directly related to resource availability (e.g. working equipments, medication, examination facilitates) [16]. This partnership was important because it demonstrated that you can empower nurses in a public, local government facility. This partnership translated into more efficient, effective health care in a comfortable environment which ensured that the patients were a priority, their needs were better met, and they were satisfied with the services.

In this study, we found that the nurses were dissatisfied working in the public clinics because the salary was low. This is a big contributor to job dissatisfaction [16]. The issue of nurse remuneration in public sectors in Uganda has been an enduring one and needs to be addressed by the MoH. To guarantee that increased remuneration produces a better product, the MoH could link portions of the salary to performance that includes measuring quality of care, resource conservation and patient satisfaction, as well as to consider non financial rewards (e.g. career development, gratitude, and recognition). The nurses in these clinics received a small additional remuneration and this may partially account for their increased satisfaction.

Burn out and intentions to leave the job are associated with increased work load as a result of a nursing shortage and a larger demand for health care [13]. Excess work load has been shown to significantly contribute to lower morale and productivity of nurses. In addition to training the staff in better time utilization this project allocated extra nurses that were deployed on project days to decreases the stress to the system. This may also account in part for the increased job satisfaction.

The knowledge and skills the nurses acquire, in part, increased their ability to provide good

nursing care. These finding agree with those of Pillay [16] who found that career opportunities and training afford individuals recognition, the prospect of further developing themselves, and growing within the ranks of their career. Nurses have more motivation and stay on the job when the environment is challenging. The nurses were also appreciative of the quality of the training they received and, in particular, noted that the training was conducted by reputable and knowledgeable people, was current information, and was structured to allow immediate implementation. For nurses in Uganda additional education is highly regarded within the context of their culture.

This study was limited by the small sample of nurses, all working in KCC clinics in an urban environment. It is difficult to clearly determine which factor or combination of factors had the largest impact and contributed to increased job satisfaction with the partnership. It could have been the small increase in salary, the increased valuing of their work, the additional education, or the better work environment. They also may have felt empowered by a broader role in the clinic, or a clearer understanding of the goals of the clinic.

## Conclusions

This community-university HIV services program was successful from the point of view of the nurses and nurse managers in improving all clinic services, and all the nurses felt successful in utilizing this opportunity for their professional advancement. This model show promise in increasing effective, quality health service; HIV and other programs can build capacity of nurses and nurse managers to directly implement such services through leadership and empowerment. The IDI has now incorporated nurse-in-charge empowerment and leadership training as key components of other PEPFAR funded HIV prevention care and treatment programs. MakCHS in their Department of Nursing has recognized the importance of educating nurses to be good managers and to be leaders in health care in Uganda and nurses in the Bachelor’s track receive specific management training. This study supports the importance of these skills to provide quality health care.

Nurses are pivotal to the effective and efficient delivery of health care in Uganda. The chronic shortages of nurses impose a real threat to the future. It therefore becomes imperative for nurse managers to identify and address those factors which are the stumbling blocks, and improve the work environment to provide a context congruent with the aspirations and value systems of nurses. This is likely to have a positive effect on individual, organizational, and population health outcomes. It also demonstrates how MakCHS can be instrumental through partnerships in designing and testing effective strategies, building human health resources and improving health outcomes in Uganda.

## List of abbreviations used

MakCHS: Makerere University College of Health Sciences; IDI: Infectious Disease Institute; KCC: Kampala City Council; MJAP: Mulago-Mbarara Teaching Hospitals’ Joint AIDS Program; HC: health center; MoH: Ministry of Health; RTC: routine HIV testing and counseling; ART: antiretroviral therapy; JHU: Johns Hopkins University; KI: key informant; VCT: Voluntary Counseling and Testing; TB: tuberculosis; PMTCT: prevention of mother-to-child transmission.

## Competing interests

The authors declare that they have no competing interests.

## Authors' contributions

NJ was involved in the conception of this paper, data analysis, and drafted the manuscript. SG was involved in the qualitative data analysis and drafting the manuscript. EL and YM contributed to the review and writing of the manuscript. NK was involved in the design of the data collection tools, ethics submissions, and contributed to the review of the manuscript.

All authors have read and approved the manuscript.
